# High Temperature Behavior of RuAl Thin Films on Piezoelectric CTGS and LGS Substrates

**DOI:** 10.3390/ma13071605

**Published:** 2020-04-01

**Authors:** Marietta Seifert

**Affiliations:** Leibniz IFW Dresden, Helmholtzstraße 20, 01069 Dresden, Germany; marietta.seifert@ifw-dresden.de; Tel.: +49-351-4659-639

**Keywords:** SAW sensors, interdigital transducer material, high temperature stability, RuAl, thin films, CTGS, LGS

## Abstract

This paper reports on a significant further improvement of the high temperature stability of RuAl thin films (110 nm) on the piezoelectric Ca${}_{3}$TaGa${}_{3}$Si${}_{2}$O${}_{14}$ (CTGS) and La${}_{3}$Ga${}_{5}$SiO${}_{14}$ (LGS) substrates. RuAl thin films with AlN or SiO${}_{2}$ cover layers and barriers to the substrate (each 20 nm), as well as a combination of both were prepared on thermally oxidized Si substrates, which serve as a reference for fundamental studies, and the piezoelectric CTGS, as well as LGS substrates. In some films, additional Al layers were added. To study their high temperature stability, the samples were annealed in air and in high vacuum up to 900 °C, and subsequently their cross-sections, phase formation, film chemistry, and electrical resistivity were analyzed. It was shown that on thermally oxidized Si substrates, all films were stable after annealing in air up to 800 °C and in high vacuum up to 900 °C. The high temperature stability of RuAl thin films on CTGS substrates was improved up to 900 °C in high vacuum by the application of a combined AlN/SiO${}_{2}$ barrier layer and up to 800 °C in air using a SiO${}_{2}$ barrier. On LGS, the films were only stable up to 600 °C in air; however, a single SiO${}_{2}$ barrier layer was sufficient to prevent oxidation during annealing at 900 °C in high vacuum.

## 1. Introduction

The development of sensors working at high temperatures is an important research field since the knowledge of process parameters at high temperatures is required to control and optimize high temperature processes. One route to realize such sensors is to use the principle of the surface acoustic waves (SAW) technology, which applies the piezoelectric effect to transfer a high frequency voltage into a mechanical wave and vice versa with electrodes that are structured with a certain geometry on a piezoelectric substrate to serve as interdigital transducers (IDTs). For the application of such sensors at high temperatures, a suitable metallization with a high thermo-mechanical stability, a low electrical resistivity, and a high oxidation and corrosion resistance, as well as a high temperature stable piezoelectric substrate are required. Several metallization systems have been investigated during the last few years concerning their high temperature stability, e.g., Pt- or Ir-based materials [1,2,3,4], oxide dispersion hardened materials [5,6], or refractory metals [7].

Another alternative material for high temperature stable electrodes is the RuAl alloy with its high melting temperature of 2050 °C [8] and strong oxidation and corrosion resistance [9]. During the last few years, we investigated the high temperature stability of RuAl thin films on the high temperature stable piezoelectric Ca${}_{3}$TaGa${}_{3}$Si${}_{2}$O${}_{14}$ (CTGS), as well as La${}_{3}$Ga${}_{5}$SiO${}_{14}$ (LGS) substrates. The experiments revealed that an oxidation barrier between the substrates and the RuAl film was required to prevent a chemical reaction between the Al and the CTGS or LGS if the samples were annealed at 800 °C in high vacuum (HV) [10,11]. It was shown that this reaction was suppressed if a 10 nm thick sputtered SiO${}_{2}$ layer was added on top of the substrate [12].

Reference RuAl films on thermally oxidized Si substrates (SiO${}_{2}$/Si) were used to study the oxidation behavior of the thin films in air, since in combination with these substrates, there is hardly any chemical reaction between film and substrate, and therefore, only a chemical reaction of the RuAl with the oxygen of the surrounding atmosphere can take place (at 800 °C, a slight reduction of the SiO${}_{2}$ to Si and the formation of Al${}_{2}$O${}_{3}$ are observed at the interface between the substrate and the RuAl film; however, this is in such a small amount that it has hardly any influence on the performance of the film). While a 20 nm thick sputtered Al${}_{2}$O${}_{3}$ cover layer fails during annealing at 800 °C in air and leads to a strong oxidation of the RuAl film, a 20 nm thick SiO${}_{2}$ cover layer successfully prevents the reaction between the film and the surrounding atmosphere at this temperature for at least 10 h [13].

However, further improvements are necessary to realize RuAl-based SAW sensors on CTGS and LGS substrates that can operate at high temperatures in ambient condition. In this paper, extended RuAl thin films in combination with barrier layer systems on CTGS and LGS substrates are investigated as a necessary step towards the production of structured IDTs. AlN thin films are tested as an alternative oxidation barrier as individual layers, as well as in combination with a SiO${}_{2}$ barrier. Former experiments showed that an increase in the Al content of the RuAl layer led to an improved high temperature stability [14]. As an alternative approach to the homogeneous increase in the Al content, 10 nm thick Al layers are added on top and below the RuAl film, and their influence on the film behavior after the annealing is analyzed.

## 2. Materials and Methods

**Figure 1:**
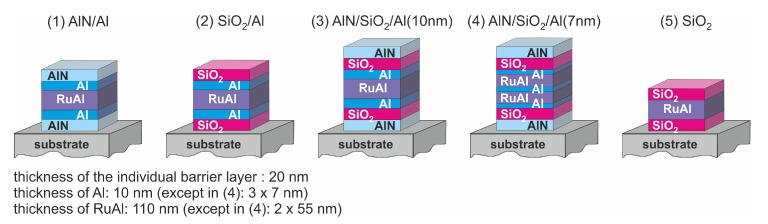
Architecture and notation of the different samples. As substrate materials thermally oxidized Si (SiO${}_{2}$/Si), CTGS and LGS are used.

RuAl thin films with a thickness of 110 nm were prepared by co-sputtering from elemental targets on Ca${}_{3}$TaGa${}_{3}$Si${}_{2}$O${}_{14}$ (CTGS) and La${}_{3}$Ga${}_{5}$SiO${}_{14}$ (LGS) substrates with either a 20 nm AlN or SiO${}_{2}$ cover and barrier layer, as well as a combination of both (total thickness 40 mm). Al layers with a thickness of 10 nm were included below and above the RuAl layer. Another layer stack consisting of 7 nm Al, 55 nm RuAl, 7 nm Al, and 55 nm RuAl covered by 7 nm Al, which was in sum again about 20 nm Al and 110 nm RuAl, was prepared as a sample with an alternative distribution of the Al. A RuAl film with a SiO${}_{2}$ cover and barrier layer without additional Al layers served as a reference sample. To reveal if oxidation processes arose from the CTGS or LGS substrate, which were known to oxidize, e.g., Al films deposited directly on these substrates, or were due to the surrounding atmosphere, the same sample systems were simultaneously deposited on thermally oxidized Si substrates (SiO${}_{2}$/Si). Details of the sputter deposition of the RuAl films were described in [10]. The AlN layers were prepared by sputter deposition from an AlN target at room temperature (RT) with a mixture of N${}_{2}$ and Ar of 1:11. SiO${}_{2}$ was deposited from a SiO${}_{2}$ target at a temperature of 180 °C with a mixture of O${}_{2}$ and Ar with a ratio of 1:6 as the sputtering gas. Figure 1 presents an overview of the analyzed samples and their notation.

The samples were annealed for 10 h at up to 900 °C in air and for comparison at 900 °C in HV. The phase formation was analyzed with X-ray diffraction in Bragg–Brentano geometry (XRD, Philips X’Pert, Co-Kα). To obtain the full texture information, pole figure measurements were performed (Philips X’Pert, Cu-Kα). Cross-sections of the samples were fabricated in a focused ion beam device (FIB, Zeiss 1540 XB CrossBeam, Carl Zeiss Microscopy GmbH, Oberkochen, Germany) and imaged by scanning electron microscopy (SEM) in the same instrument. SEM images of the surfaces of the samples were obtained with a Zeiss Ultra Plus (Carl Zeiss Microscopy GmbH, Oberkochen, Germany).

The distribution of the elements across the sample thickness was determined by Auger electron spectroscopy (AES, JEOL JAMP-9500F Field Emission Auger Microprobe). AES depth profiles were realized by alternately sputtering with Ar ions with an energy of 1 kV for 120 s and measuring of the AES spectra. High resolution images of the sample cross-sections were recorded by annular dark field scanning transmission electron microscopy (ADF-STEM, Tecnai F30, FEI company, Hillsboro, OR, USA) in combination with energy dispersive X-ray spectroscopy (EDX, Octane T Optima, EDAX Company, Mahwah, NJ, USA) to reveal the local chemical composition.

The electrical resistivity of the films was determined by the van der Pauw technique (vdPauw, W tips). An electrical current of 5 mA was injected into the sample, and its polarity was changed after each measurement. A nanovoltmeter (2182A-Nanovoltmeter, KEITHLEY-TEKTRONIX Inc., Beaverton, OR, USA) was used to measure the voltage. The electrical resistivity was calculated from the measured resistance and the sample thickness.

## 3. Results and Discussion

### 3.1. Film Morphology

**Figure 2:**
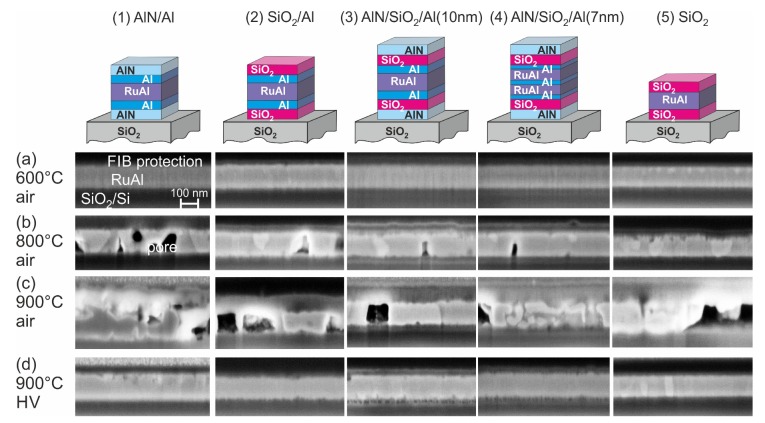
SEM images (inLens, 3 kV) of cross-sections of the RuAl thin films with various protection layers on SiO${}_{2}$/Si after annealing in air for 10 h at (**a**) 600 °C, (**b**) 800 °C, (**c**) 900 °C, and (**d**) in high vacuum at 900 °C.

**Figure 3:**
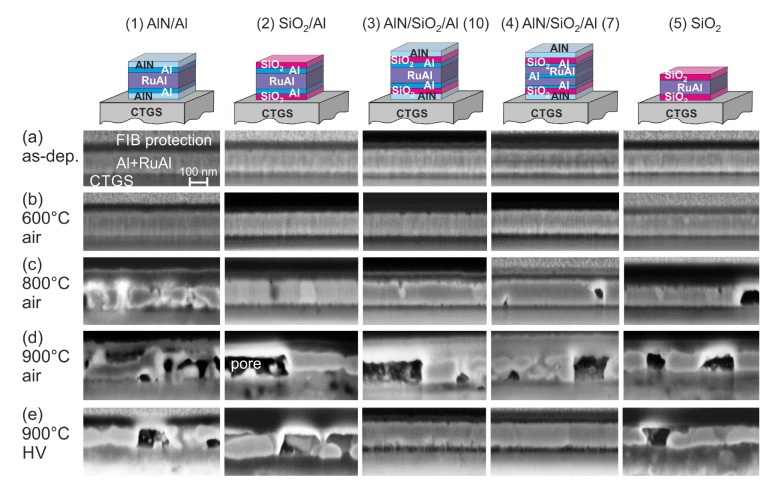
SEM images (inLens, 3 kV) of cross-sections of the RuAl thin films with various protection layers on CTGS in (**a**) the as-deposited state and after annealing in air for 10 h at (**b**) 600 °C, (**c**) 800 °C, (**d**) 900 °C, and (**e**) in high vacuum at 900 °C.

**Figure 4:**
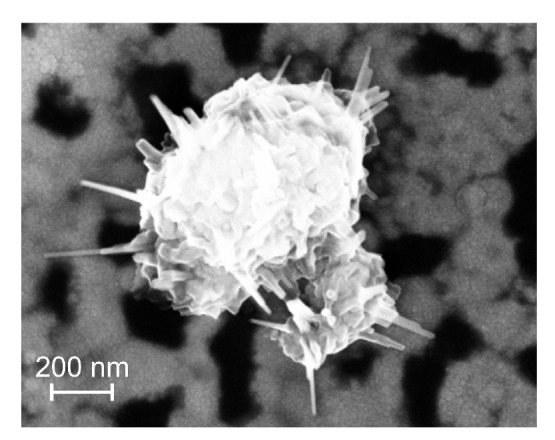
SEM image (inLens, 3 kV) of the surface of the sample with the (5)-SiO${}_{2}$ barrier layer on CTGS at a position with a defect.

**Figure 5:**
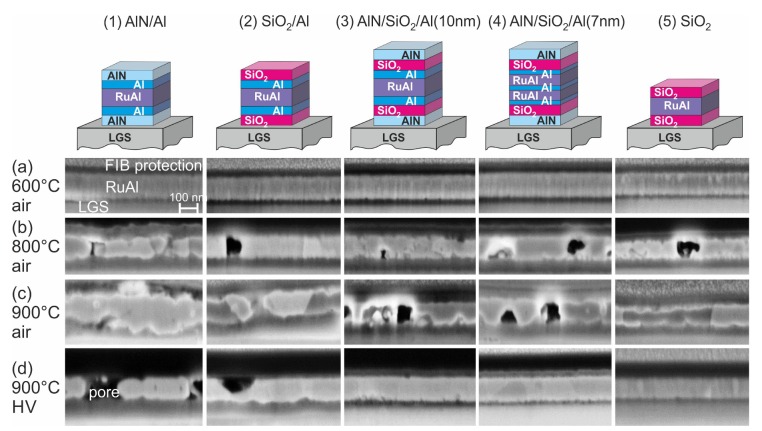
SEM images (inLens, 3 kV) of cross-sections of the RuAl thin films with various protection layers on LGS after annealing in air for 10 h at (**a**) 600 °C, (**b**) 800 °C, (**c**) 900 °C, and (**d**) in high vacuum at 900 °C.

To analyze the high temperature stability and possible reactions of the sample with the substrate and the surrounding atmosphere, cross-section images of the substrate-sample systems were evaluated. The SEM images of the cross-sections prepared with FIB for the films on SiO${}_{2}$/Si, CTGS, and LGS, respectively, together with an exemplary SEM image of the surface of a sample on CTGS are summarized in Figure 2, Figure 3, Figure 4 and Figure 5.

After heat treatment at 600 °C in air, the samples on SiO${}_{2}$/Si were not degraded (Figure 2a). Only the sample with SiO${}_{2}$ barriers without additional Al layers ((5)-SiO${}_{2}$) showed some oxide formation, which is visible from the small bright grains at the surface, which consist of Ru, as shown in former work. Such Ru-rich grains are formed if Al is oxidized and lacks for the formation of the RuAl phase. The sample morphology strongly changed after heat treatment at 800 °C in air (Figure 2b). The (1)-AlN/Al sample contained many pores, which indicates strong oxidation effects. The (2)-SiO${}_{2}$/Al, (3)-AlN/SiO${}_{2}$/Al(10 nm), and (4)-AlN/SiO${}_{2}$/Al(7 nm) samples were still intact and only contained small pores. The (5)-SiO${}_{2}$ sample was also still intact; however, showing a rough surface.

The heat treatment at 900 °C in air led to a strong degradation of all samples (Figure 2c). In the films with a single barrier, it can be seen that there is no clear interface to the substrate any more. This indicates a strong interaction between the film and the substrate material. The films with the combined barrier layers were slightly less oxidized as compared to the other samples.

After annealing at 900 °C in HV, some small brighter grains were only visible for the (1)-AlN/Al sample at the film surface, which again indicates a partial oxidation. Besides this, none of the samples were degraded (Figure 2d).

The air annealed samples on CTGS (as-prepared state shown in Figure 3a) behaved like the corresponding samples on the thermally oxidized Si substrates. All samples were intact after annealing at 600 °C in air (Figure 3b). The 800 °C annealing in air led to a degradation of the film with AlN barrier, while the other samples were not damaged, and only small pores were visible (Figure 3c). After annealing at 900 °C in air, all samples were strongly degraded (Figure 3d). However, damages in the CTGS substrate were only present for both samples with the SiO${}_{2}$ barrier with and without additional Al layers. SEM images of the surface of the samples revealed the formation of bulges for all systems. Such bulges were found for the (1)-AlN/Al system already for the annealings at a lower temperature in air. On the sample surface of the films annealed at 900 °C in air, locally, structures with sharp needles formed, as shown in Figure 4. An EDX analysis revealed that these structures mainly consisted of Ga oxides, Ga-Al oxides, and Si oxides.

The influence of the CTGS substrate became visible in the samples annealed at 900 °C in high vacuum (Figure 3e). While all samples on SiO${}_{2}$/Si were stable after this annealing, on CTGS, the (1)-AlN/Al, (2)-SiO${}_{2}$/Al, and (5)-SiO${}_{2}$ samples were oxidized. The film with the SiO${}_{2}$ barrier without additional Al layers appeared less oxidized as compared to the film with the additional Al layers. The images also show a degradation of the surface of the CTGS substrate. Only the two samples with the combined barrier layers (3)-AlN/SiO${}_{2}$/Al(10 nm) and (4)-AlN/SiO${}_{2}$/Al(7 nm) were not oxidized. Although a 10 nm thick SiO${}_{2}$ barrier layer on top of the CTGS substrate was sufficient to prevent the diffusion of oxygen out of the substrate into the RuAl film if the sample was annealed at 800 °C in HV [12], at 900 °C, a SiO${}_{2}$ film with even twice the thickness was not sufficient.

Figure 5 summarizes the cross-section images of the films on the LGS substrates. For this kind of substrate also, the annealing at 600 °C in air did not degrade the films (Figure 5a). However, in contrast to the films on the other substrates after annealing at 800 °C in air, all films were strongly oxidized and peeled off the substrate to a large extent. The cross-section images show regions of the films that were still in contact with the LGS (Figure 5b). Only the films with the SiO${}_{2}$ barrier were still attached to a large extent to the substrate. The (4)-AlN/SiO${}_{2}$/Al(7 nm) sample was the most destroyed film with hardly any film in contact with the substrate. However, in contrast to the films with CTGS, no inhomogeneities were observed in the surface region of the substrate.

The annealing at 900 °C in air likewise led to a strong degradation of the films (Figure 5c) and to a large-scale peeling of the film off the substrate. Again, the cross-section images represent regions of the film that were still attached to the LGS.

The annealing at 900 °C in HV led to a strong degradation of the (1)-AlN/Al sample (Figure 5d), and locally, defect formation in the LGS substrate up to a depth of a few μm was observed. For all other samples, the RuAl layer was still visible. The presence of the additional Al layers in the sample with SiO${}_{2}$ barriers resulted in a stronger chemical reaction between the film and the LGS substrate, which was visible from the irregular rough interface between the film and the substrate, while in the sample without additional Al, still, a smooth interface was present. There was also a local degradation of the LGS for the (2)-SiO${}_{2}$/Al sample.

The formation of bulges in the film was observed for almost all annealed samples on LGS.

### 3.2. Phase Formation

**Figure 6:**
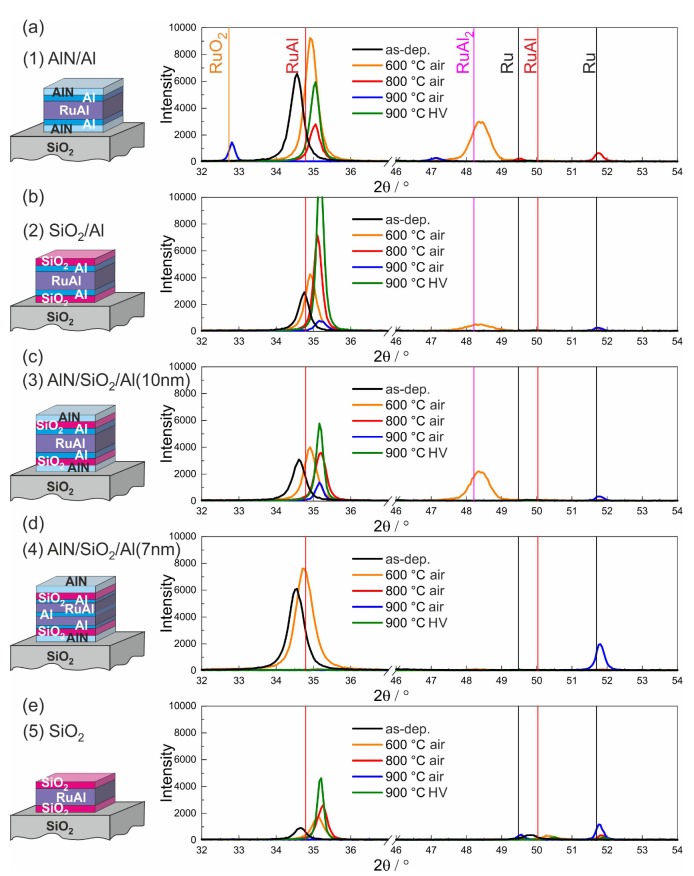
Results of the XRD measurements (Bragg–Brentano, Co-Kα) for the samples with the different cover and barrier layers on SiO${}_{2}$/Si substrates: (**a**) (1)-AlN/Al, (**b**) (2)-SiO${}_{2}$/Al, (**c**) (3)-AlN/SiO${}_{2}$/Al(10 nm), (**d**) (4)-AlN/SiO${}_{2}$/Al(7 nm), and (**e**) (5)-SiO${}_{2}$.

**Figure 7:**
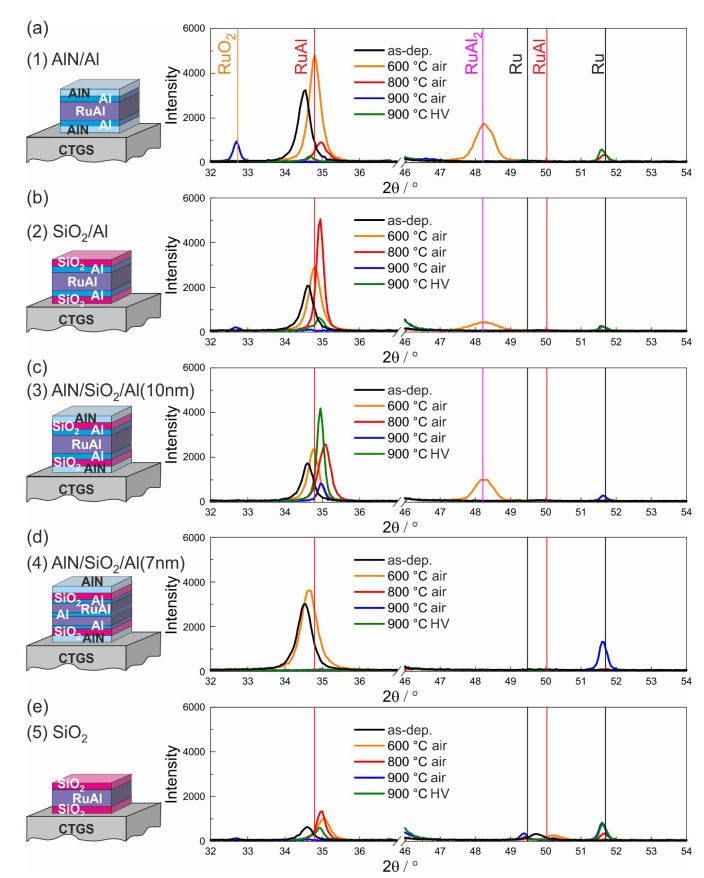
Results of the XRD measurements (Bragg–Brentano, Co-Kα) for the samples with the different cover and barrier layers on CTGS substrates: (**a**) (1)-AlN/Al, (**b**) (2)-SiO${}_{2}$/Al, (**c**) (3)-AlN/SiO${}_{2}$/Al(10 nm), (**d**) (4)-AlN/SiO${}_{2}$/Al(7 nm), and (**e**) (5)-SiO${}_{2}$.

**Figure 8:**
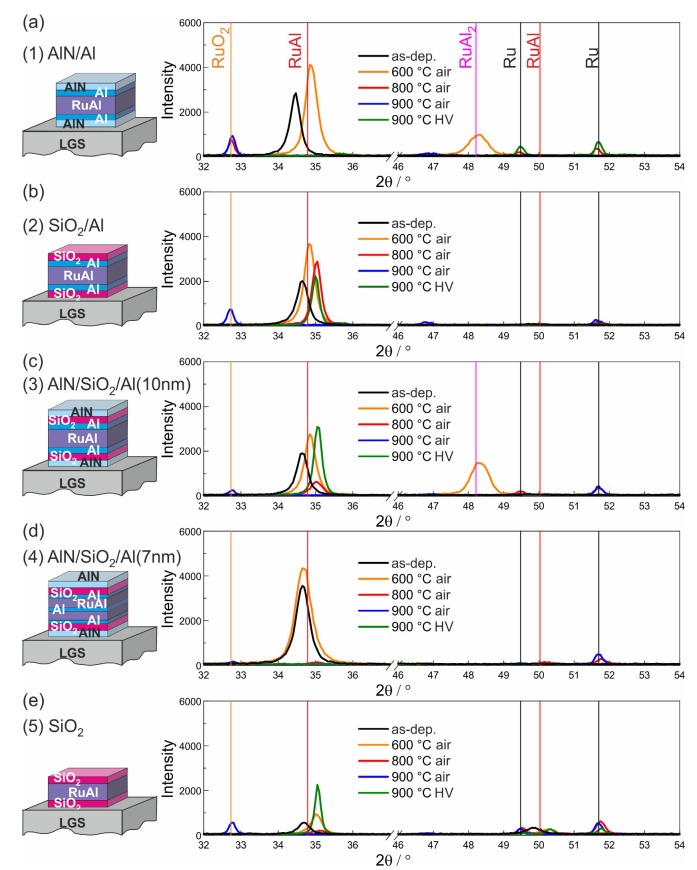
Results of the XRD measurements (Bragg–Brentano, Co-Kα) for the samples with the different cover and barrier layers on LGS substrates: (**a**) (1)-AlN/Al, (**b**) (2)-SiO${}_{2}$/Al, (**c**) (3)-AlN/SiO${}_{2}$/Al(10 nm), (**d**) (4)-AlN/SiO${}_{2}$/Al(7 nm), and (**e**) (5)-SiO${}_{2}$.

The XRD measurements of the different film systems on SiO${}_{2}$/Si, CTGS, and LGS are presented in Figure 6, Figure 7 and Figure 8.

All samples deposited on SiO${}_{2}$/Si showed a (100) RuAl peak in the as-deposited state, which was located at a slightly lower angle (shift up to −0.23°) as compared to the bulk RuAl value (2θ=34.8°). Annealing at 600 °C in air led to an increase in the intensity of the RuAl (100) peak for all samples and to a shift to higher 2θ values than the theoretical position of the RuAl (100) (up to 0.36°) except for the (4)-AlN/SiO${}_{2}$/Al(7 nm) film, which showed the peak at 34.74°. In the case of the (1)-AlN/Al (Figure 6a) and (3)-AlN/SiO${}_{2}$/Al(10 nm) (Figure 6c) samples, a RuAl${}_{2}$ peak became visible at a 2θ position of 48.4°, which is slightly shifted with respect to the bulk value of the RuAl${}_{2}$ (004) reflex (2θ=48.3°). A very small RuAl${}_{2}$ peak also appeared for the (2)-SiO${}_{2}$/Al sample (Figure 6b).

The heat treatment at 800 °C in air resulted in a further shift of the RuAl (100) peak to higher 2θ values (up to 0.46°) and the disappearance of the RuAl${}_{2}$ peak. In the case of the (1)-AlN/Al sample, the RuAl (100) peak intensity strongly decreased, which is in accordance with the strong degradation, which was visible in the cross section image (Figure 2b). The oxidation of Al also explains the appearance of Ru peaks. The RuAl peak intensity was slightly reduced for the (3)-AlN/SiO${}_{2}$/Al(10 nm) film and was increased for both films with the SiO${}_{2}$ barrier. Surprisingly, in the case of the (4)-AlN/SiO${}_{2}$/Al(7 nm) film, no RuAl peak was visible any more, also for higher annealing temperatures, although the cross-section images were comparable to the (3)-AlN/SiO${}_{2}$/Al(10 nm) sample and did not show a stronger degradation.

After annealing at 900 °C in air, the RuAl peak disappeared for the (1)-AlN/Al and (5)-SiO${}_{2}$ sample. For the (1)-AlN/Al sample, a RuO${}_{2}$ (110) peak at 2θ of 32.7° appeared. The (2)-SiO${}_{2}$/Al and (3)-AlN/SiO${}_{2}$/Al(10 nm) sample still showed a small RuAl peak, and a very small Ru peak became visible. The AlN/SiO${}_{2}$/Al(7 nm) showed a stronger Ru peak.

Annealing in high vacuum at 900 °C resulted in a RuAl peak that was higher than for the other annealing procedures in the case of the (2)-SiO${}_{2}$/Al, (3)-AlN/SiO${}_{2}$/Al(10 nm), and (5)-SiO${}_{2}$ samples. The (1)-AlN/Al sample showed a RuAl peak with a reduced intensity as compared to the as-prepared state.

In summary, the XRD measurements confirmed that AlN is not a suitable barrier layer for RuAl thin films. Comparing the samples (2)-SiO${}_{2}$/Al and (5)-SiO${}_{2}$ revealed that the additional Al layers are beneficial for the RuAl phase formation. A comparison between the samples (3)-AlN/SiO${}_{2}$/Al(10 nm) and (4)-AlN/SiO${}_{2}$/Al(7 nm) showed that despite a similar film morphology visible from the cross-section images, there is a strong difference in phase formation. This issue will be discussed below.

The formation of the RuAl${}_{2}$ phase during the annealing at 600 °C was the result of the interdiffusion between the Al and RuAl layers. At the interface, a region with a higher Al content developed, which led to the formation of the Al rich RuAl${}_{2}$ phase. A comparison between the (3)-AlN/SiO${}_{2}$/Al(10 nm) and (4)-AlN/SiO${}_{2}$/Al(7 nm) films indicated that the reduced thickness of the Al layer of 7 nm was not sufficient to realize the formation of RuAl${}_{2}$. In both films, the same total amount of Al was added so that the absence of the RuAl${}_{2}$ in the (4)-AlN/SiO${}_{2}$/Al(7 nm) film can only be explained by the different local distribution. During the annealing at higher temperatures, two processes took place: The further interdiffusion of the Al within the RuAl film led to a lower local concentration of the Al. On the other hand, first oxidation processes took place, which also reduced the Al concentration, so that the Al content was not sufficient to form the RuAl${}_{2}$ phase any more.

The results of the XRD measurements of the films on the CTGS substrates are shown in Figure 7. As for the films on SiO${}_{2}$/Si, all films showed the RuAl (100) peak at a slightly lower 2θ value in the as-prepared state. Annealing at 600 °C in air led to an increase in peak intensity and to a shift to higher 2θ values for all samples. In this case also, the RuAl${}_{2}$ phase was formed for the (1)-AlN/Al and (3)-AlN/SiO${}_{2}$/Al(10 nm) films and with a very low amount also for the (2)-SiO${}_{2}$/Al film. Furthermore, the behavior after annealing at 800 °C in air was similar to that of the films on the thermally oxidized Si substrates: the peak intensity was increased for the films with the SiO${}_{2}$ barrier, slightly increased for the (3)-AlN/SiO${}_{2}$/Al(10 nm) sample, and strongly reduced for the (1)-AlN/Al film. Again, no RuAl peaks were measured for the (4)-AlN/SiO${}_{2}$/Al(7 nm) samples for 800 °C and higher temperatures.

Differences of the films on CTGS to the samples on the thermally oxidized Si substrates became obvious for the samples annealed at 900 °C in air and in HV. For the samples annealed in air, only for the (3)-AlN/SiO${}_{2}$/Al(10 nm) film, a small RuAl reflex remained. For the samples on SiO${}_{2}$/Si, RuAl was additionally still visible for the (2)-SiO${}_{2}$/Al film. While for all samples on the SiO${}_{2}$/Si substrate annealed at 900 °C in HV, a strong RuAl peak was measured, in the case of the CTGS substrate, a strong RuAl peak was only visible for the (3)-AlN/SiO${}_{2}$/Al(10 nm) sample system, and only very small peaks appeared for both films with the SiO${}_{2}$ barrier.

Figure 8 summarizes the results of the XRD measurements of the different films on the LGS substrates. As for the other two systems for all films, a RuAl peak was visible in the as-prepared state. The intensity increased after annealing at 600 °C in air, and a RuAl${}_{2}$ peak appeared for the (1)-AlN/Al and (3)-AlN/SiO${}_{2}$/Al(10 nm) sample system. However, after annealing at 800 °C in air, where for the other two substrates, RuAl was visible for all films, with decreased intensity for the (1)-AlN/Al sample, in the case of LGS, the RuAl peak was only visible for the (2)-SiO${}_{2}$/Si and with a strongly reduced intensity for the (3)-AlN/SiO${}_{2}$/Al(10 nm) layer stack. The film with the SiO${}_{2}$ barrier without the additional Al layer did not show RuAl anymore. For the film with the AlN barrier, already at this temperature, RuO${}_{2}$ was formed, which was not detected in any of the films on the other substrates.

After annealing at 900 °C in air, for none of the samples RuAl was measured. Instead, besides the (4)-AlN/SiO${}_{2}$/Al(7 nm) sample, all films showed the formation of RuO${}_{2}$.

These results revealed that there is an improved high temperature stability of CTGS as compared to LGS concerning heat treatments in air. On the other hand, the films on LGS showed a stronger phase formation and an improved film stability after annealing at 900 °C in high vacuum. In the case of LGS, a strong RuAl peak was not only measured for the (3)-AlN/SiO${}_{2}$/Al(10 nm) layer stack, as was the case for the CTGS substrate, but also for both samples with the SiO${}_{2}$ barrier without and with the additional Al layers. The better stability under high vacuum conditions was already visible in the cross-section images in Figure 5, which showed a more homogeneous film structure and less reactions with the substrate for these samples.

**Figure 9:**
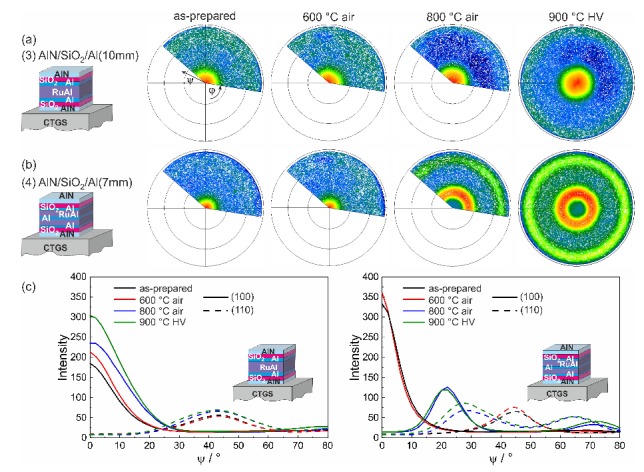
Pole figure measurements of the RuAl (100) pole for (**a**) the system (3)-AlN/SiO${}_{2}$/Al(10 nm) and (**b**) the system (4)-AlN/SiO${}_{2}$/Al(7 nm) on CTGS in the as-prepared state and after annealing at 600 and 800 °C in air and 900 °C in high vacuum. (**c**) shows the averaged intensity of the (100) and (110) pole figures dependent on the tilt angle ψ for both systems.

Common for all substrates is the different behavior of both systems with the combined barrier layers with the varying distribution of the Al. While the layer stack (3)-AlN/SiO${}_{2}$/Al(10 nm) showed a RuAl peak for all annealing conditions (except for LGS, where no peak was visible for the 900 °C air annealed sample), in the case of the (4)-AlN/SiO${}_{2}$/Al(7 nm) sample, the RuAl peak was measured only for the as-prepared and 600 °C annealed sample. For the evaluation of these data, however, one has to be aware of the fact that the measurements in Bragg–Brentano geometry are only sensitive to lattice planes, which are oriented parallel to the film surface. To reveal the whole texture information, pole figure measurements were conducted. The pole figures of the (100) RuAl peak for the as-prepared state and after annealing at 600 and 800 °C in air and 900 °C in HV are presented in Figure 9a,b for the (3)-AlN/SiO${}_{2}$/Al(10 nm) and (4)-AlN/SiO${}_{2}$/Al(7 nm) systems, using the example of the films on CTGS. In addition, the RuAl (110) pole was measured.

For both layer systems, the pole figures revealed a (100) fiber texture in the as-prepared state and after annealing at 600 °C in air. While this (100) fiber texture was maintained in the case of the (3)-AlN/SiO${}_{2}$/Al(10 nm) sample for the heat treatments at higher temperatures (Figure 9a), a change in the orientation of the texture appeared in the (4)-AlN/SiO${}_{2}$/Al(7 nm) system (Figure 9b). The pole figure showed a fiber texture with a tilt of the (100) planes of 22° with respect to the film normal, and the corresponding second ring of the fiber texture appeared at a ψ of 74°. These results explain why no intensity was measured in the Bragg–Brentano XRD: the measured intensity in this case corresponded to the intensity at ψ=0 (no tilt of the sample) in the pole figures.

The images of the pole figures are scaled with respect to their maximum intensity. To allow a comparison between the textures of the samples, in Figure 9c the azimuthal averaged intensity versus the tilt angle ψ is plotted for the (100) and the (110) pole. In case of the (3)-AlN/SiO${}_{2}$/Al(10 nm) sample, the highest intensity was achieved for the sample annealed at 900 °C in HV, which is in agreement with the XRD results obtained in Bragg–Brentano geometry. The half width at half maximum increased with increasing annealing temperature, which means that the quality of the texture decreased. The plot for the (4)-AlN/SiO${}_{2}$/Al(7 nm) system illustrates the similar texture for the as-prepared and 600 °C sample. There is a better quality of the texture as compared to the other layer stack. The plots of the samples annealed at the higher temperatures clearly demonstrated the tilt of the lattice planes with almost the same quality of texture.

To conclude, the XRD measurements in Bragg–Brentano geometry and the pole figure measurements revealed the following findings:

- The presence of additional Al layers improves the RuAl phase formation. This became obvious comparing the sample systems (2)-SiO${}_{2}$/Al and (5)-SiO${}_{2}$.
- Changing the distribution of Al within the layer system can change the texture of the RuAl phase after annealing at higher temperatures, leading to a tilted orientation of the (100) lattice planes.

### 3.3. Film Chemistry

**Figure 10:**
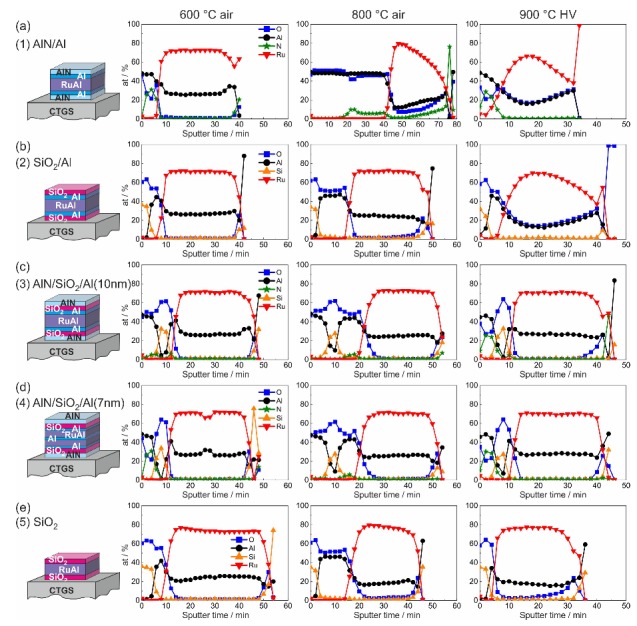
Results of the AES measurements for the samples with the different cover and barrier layers on CTGS substrates after annealing at 600 and 800 °C in air and 900 °C in HV: (**a**) (1)-AlN/Al, (**b**) (2)-SiO${}_{2}$/Al, (**c**) (3)-AlN/SiO${}_{2}$/Al(10 nm), (**d**) (4)-AlN/SiO${}_{2}$/Al(7 nm), and (**e**) (5)-SiO${}_{2}$.

Figure 10 summarizes the results of the AES depth profile measurements of the samples after the heat treatment at 600 and 800 °C in air and at 900 °C in HV. Since the CTGS substrates are most promising for future applications in high temperature sensors, the AES measurements were conducted for these substrates. The evaluation of the AES was done using standard relative sensitivity factors. Together with the effect of a preferential sputtering, the calculations of the atomic concentration of the RuAl phase pretended a much higher Ru content. Therefore, the AES measurements are only suited to compare the samples and to follow the oxidation processes. Due to charging effects, it was not possible to measure the barrier layers at the interface to the substrate and the insulating CTGS itself.

After annealing at 600 °C in air, all samples showed an intact RuAl layer. The measurement of the (1)-AlN/Al layer (Figure 10a) revealed that the cover layer consisted of Al, N, and O. Already in the as-prepared state, the AlN layer contained some O (about 20 at% according to the AES evaluation). Despite the presence of O, we denote this layer material as AlN. In both films with the SiO${}_{2}$ cover layer with (Figure 10b) and without (Figure 10e) the additional Al layer, an Al${}_{2}$O${}_{3}$ layer formed below the SiO${}_{2}$. Its thickness was independent of the presence of the additional Al. However, in the case of the film with additional Al, a constant concentration of Ru and Al across the RuAl film thickness was observed, while in case of the film without the additional Al, the Ru content was slightly higher at the surface and was slowly reduced approaching the substrate; correspondingly, the Al content increased.

The films with the combined cover/barrier showed only a small oxidation of the Al at the film surface for the (3)-AlN/SiO${}_{2}$/Al(10 nm) system and hardly any oxidation for the (4)-AlN/SiO${}_{2}$/Al(7 nm) sample (Figure 10c,d). For the sample with the intermediate Al layer, an Al maximum was visible in the center of the RuAl film, which shows that the interdiffusion of the Al was not completed at this temperature. Regarding the distribution of the N in the cover layers, a difference was visible between both systems: while for the (4)-AlN/SiO${}_{2}$/Al(7 nm) sample, a clear N signal was measured in the uppermost layer, the N signal was very low in the (3)-AlN/SiO${}_{2}$/Al(10 nm) system, and a low N concentration was measured in the SiO${}_{2}$ layer.

The time needed to sputter the RuAl film was comparable for all films with the additional Al layers. In contrast to this, the sputter time was about 25% longer for the film without additional Al (Figure 10e).

The heat treatment at 800 °C in air led to a strong oxidation of the sample with the AlN cover/barrier layer (Figure 10a), which was already visible in the cross-section image (Figure 3b). Al${}_{2}$O${}_{3}$ was formed at the sample surface, followed by a layer consisting of Al, O, and a small content of N. In the lower region of the film Ru, Al, O, and N were measured. According to the XRD measurements, the sample contained RuAl, as well as Ru grains. Therefore, the measured AES signal can be explained by a superposition of RuAl, Ru, and Al${}_{2}$O${}_{3}$ grains (Al${}_{2}$O${}_{3}$ grew with an amorphous structure and was not visible in the XRD) and a partial diffusion of N from the lower barrier into the overlying film.

In both samples with SiO${}_{2}$ barrier layers, the thickness of the Al${}_{2}$O${}_{3}$ layer below the SiO${}_{2}$ increased and was the same for both systems. However, the Ru concentration in the RuAl layer was slightly higher in the film without the additional Al layers (Figure 10e) and slightly decreased approaching the substrate.

The AES measurements of both samples with the combined barriers were similar despite the different initial distribution of the additional Al. An Al${}_{2}$O${}_{3}$ layer was now present at the sample surface, followed by a SiO${}_{2}$ layer and another Al${}_{2}$O${}_{3}$ layer, which contained some N. In contrast to the film annealed at 600 °C in air, the annealing at 800 °C was sufficient to realize a homogeneous interdiffusion of the Al into the RuAl film.

These results demonstrated that even for the combined barrier layers, a partial diffusion of O into the RuAl layer took place during the annealing at 800 °C in air. However, the time that was necessary to sputter the RuAl layer was only slightly reduced, indicating almost the same thickness of the RuAl layer (small deviations in sputtering time can also arise from the sputtering process itself).

After annealing at 900 °C in high vacuum, the film with the AlN or SiO${}_{2}$ cover/barrier with the additional Al layers showed the presence of oxygen across the whole film. Already in the cross-section images, these samples showed a clear morphology change and the formation of pores. The sample (5)-SiO${}_{2}$ without the additional Al was significantly less oxidized as the corresponding sample with Al. This film showed only a thin Al${}_{2}$O${}_{3}$ layer below the SiO${}_{2}$ cover layer and just a slight increase in O approaching the interface to the substrate (Figure 10e). This increase in O may arise from a simultaneous sputtering of the RuAl film and the O rich substrate because of the presence of pores in the film.

Both films with the combined cover/barrier layer did not contain O in the RuAl layer, and only a very thin Al${}_{2}$O${}_{3}$ layer formed below the cover layers. In addition, it was obvious that the barrier layers at the surface of the film were still visible as AlN followed by SiO${}_{2}$, which was in contrast to the films annealed at 800 °C in air where hardly any N was detected and a three layer system Al${}_{2}$O${}_{3}$–SiO${}_{2}$–Al${}_{2}$O${}_{3}$ was formed. The systems with the combined barrier layers showed a difference at the interface to the lower barrier layers. In the case of the (3)-AlN/SiO${}_{2}$/Al(10 nm) sample, there was a stronger increase in O in the RuAl film, indicating a partial oxidation of Al to Al${}_{2}$O${}_{3}$, which was more pronounced as compared to the (4)-AlN/SiO${}_{2}$/Al(7 nm) layer stack.

### 3.4. TEM Studies on Layer Stacks (3) and (4)

In the cross-section images and AES measurements, hardly any differences between the layer stacks (3)-AlN/SiO${}_{2}$/Al(10 nm) and (4)-AlN/SiO${}_{2}$/Al(7 nm) were visible. To analyze more in detail the local morphology and chemistry, STEM measurements in combination with EDX were performed. These measurements also allowed analyzing the barrier layers between the film and the CTGS substrate, which cannot not be measured in AES due to the charging effects.

**Figure 11:**
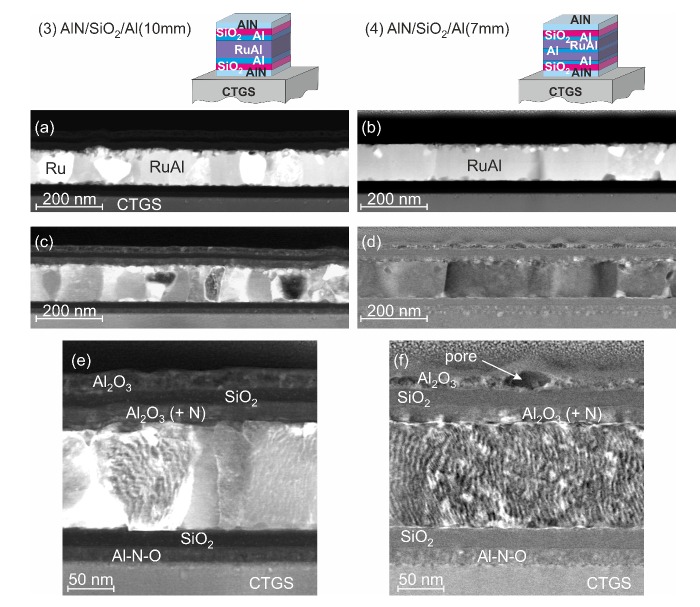
STEM images of layer stacks (3)-AlN/SiO${}_{2}$/Al(10 nm) and (4)-AlN/SiO${}_{2}$/Al(7 nm) on CTGS after annealing at 800 °C in air: (**a**,**b**) with predominant chemical contrast and (**c**–**f**) with predominant orientation contrast.

Figure 11 shows the images for the samples after annealing at 800 °C in air. These results revealed that there were differences between both sample systems. The images with predominant chemical contrast (Figure 11a,b) showed that the (4)-AlN/SiO${}_{2}$/Al(7 nm) film had a much more homogeneous structure with significantly less Ru rich grains.

In the images with predominant orientation contrast, it could be seen that in the (3)-AlN/SiO${}_{2}$/Al(10 nm) sample, the grains were mostly extended across the thickness of the layer and had an in-plane grain size between a few tens and about 100 nm (Figure 11c). In the (4)-AlN/SiO${}_{2}$/Al(7 nm) sample, all grains were extended across the whole film thickness and had an in-plane grain size up to several 100 nm (Figure 11d). The images with higher magnification (Figure 11e,f) showed that the structure of the cover layers was also slightly different. In the (3)-AlN/SiO${}_{2}$/Al(10 nm) sample, a three layer structure consisting of a Al${}_{2}$O${}_{3}$ layer on top, followed by a SiO${}_{2}$ layer, and finally, another Al${}_{2}$O${}_{3}$ layer, which contained a small amount of N, was visible, which was already derived from the AES measurements. Although this three layer structure was likewise measured in AES for the (4)-AlN/SiO${}_{2}$/Al(7 nm) layer stack, the STEM measurements revealed that the top Al${}_{2}$O${}_{3}$ layer had a higher roughness and contained pores.

At the interface to the substrate, the initial order of the barrier layers (the AlN (AlNO) layer on top of the CTGS followed by SiO${}_{2}$) was kept for both samples, and there were hardly any inhomogeneities in the surface region of the CTGS.

**Figure 12:**
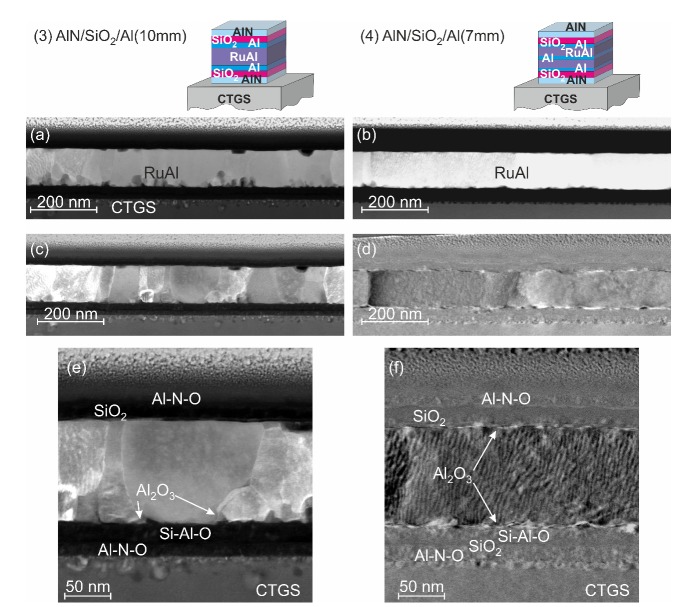
STEM images of layer stacks (3)-AlN/SiO${}_{2}$/Al(10 nm) and (4)-AlN/SiO${}_{2}$/Al(7 nm) on CTGS after annealing at 900 °C in HV: (**a**,**b**) with predominant chemical contrast; (**c**–**f**) with predominant orientation contrast.

The STEM images of the samples after annealing at 900 °C in HV are summarized in Figure 12. For both kinds of samples, the images with predominant chemical contrast showed a more homogeneous structure as compared to the samples annealed at 800 °C in air. However, for the (3)-AlN/SiO${}_{2}$/Al(10 nm) layer stack, inhomogeneities were visible in the RuAl film at the lower interface (Figure 12a). These darker regions represent Al${}_{2}$O${}_{3}$ grains. For this kind of sample, inhomogeneities were present in the upper region (about 50 nm) of the CTGS (Figure 12a,e).

In the case of the (4)-AlN/SiO${}_{2}$/Al(7 nm) sample, a very thin layer (a few nm) of Al${}_{2}$O${}_{3}$ was formed at the interface between the RuAl and upper and lower SiO${}_{2}$ layer (Figure 12f). There were significantly less inhomogeneities in the upper region of the CTGS as compared to the other system. The stronger oxidation at the interface to the substrate of the (3)-AlN/SiO${}_{2}$/Al(10 nm) film as compared to the (4)-AlN/SiO${}_{2}$/Al(7 nm) layer stack was already derived from the AES measurements. This difference in oxidation is in agreement with the different degrees of degradation of the CTGS at the interface to the film.

Regarding the film morphology, it was again obvious that the grain structure was more inhomogeneous in the (3)-AlN/SiO${}_{2}$/Al(10 nm) system, and there were grains that were not extended across the film thickness. The grain size distribution ranged from a few 10 nm up to 200 nm. Again, the grains in the (4)-AlN/SiO${}_{2}$/Al(7 nm) sample had a much larger lateral expansion (≈ 100–500 nm).

The EDX analysis of the barrier and cover layers revealed that during the HV annealing, the upper barriers remained unchanged, so that there was still the AlN-SiO${}_{2}$ bilayer. However, in the lower barrier, there was an interdiffusion of Al into the SiO${}_{2}$. In the case of the (3)-AlN/SiO${}_{2}$/Al(10 nm) system, Al was distributed homogeneously in the SiO${}_{2}$ layer, while in the (4)-AlN/SiO${}_{2}$/Al(7 nm) sample, Al was only found in the upper region of the SiO${}_{2}$ layer, and the lower region was free of Al.

In summary, the STEM investigations revealed differences in the film morphology between the (3)-AlN/SiO${}_{2}$/Al(10 nm) and (4)-AlN/SiO${}_{2}$/Al(7 nm) sample systems. The latter was more homogeneous with larger grains and less oxidation effects at the boundaries of the RuAl film.

### 3.5. Electrical Properties

**Figure 13:**
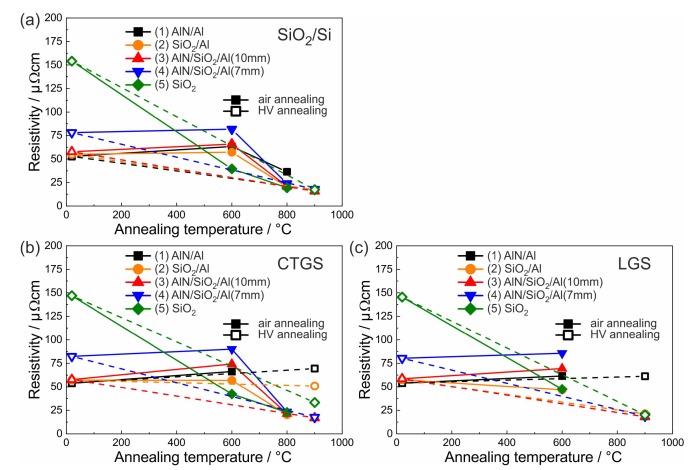
Electrical resistivity of the different film systems on (**a**) SiO${}_{2}$/Si, (**b**) CTGS, and (**c**) LGS substrates in the as-prepared state and after annealing. For SiO${}_{2}$/Si and CTGS, the values for the air annealed samples are shown up to 800 °C and for LGS up to 600 °C. The full symbols represent the annealing in air, the open symbols in HV. The straight lines visualize samples of the same set.

For the application as SAW electrodes, the electrical resistivity $R_{el}$ of the metallization has to be known to optimize the device layout. The sheet resistance of the films on the different substrates was determined by the van der Pauw method, and then, the electrical resistivity was calculated using the film thickness. The thickness of the RuAl and Al layers, however, was only known for the as-deposited state, and the effective thickness of the conductive layer after the heat treatments was reduced due to the oxidation effects. Despite this, the initial thickness (130 nm for the film systems (1)–(4) and 110 nm for the film system (5)) was used to calculate the resistivity, which meant that the actual resistivity of the annealed films was slightly lower. Figure 13 summarizes the values of the electrical resistivity for the films on the different substrates.

In the as-prepared state, $R_{el}$ was the lowest for the films with the two times 10 nm additional Al layers and hardly depended on the applied substrate and barrier/cover layers (53–59 μΩcm range for the different substrates and barrier layers, deviation less than 15 %). The (4)-AlN/SiO${}_{2}$/Al(7 nm) films had a slightly higher $R_{el}$ (78–82 μΩcm), and the film without additional Al showed the highest $R_{el}$ of 145–154 μΩcm. These results proved that in the as-prepared state, the electrical resistivity was dominated by the Al layers with their excellent electrical properties. Two thicker Al films led to a lower resistivity than three Al layers with the same total thickness due to an increased scattering of the electrons because of the reduced film thickness and at the additional two interfaces between the Al and the RuAl.

After annealing at 900 °C in HV, all films on the SiO${}_{2}$/Si substrates had almost the same $R_{el}$ (16–18 μΩcm), which was much lower as compared to the as-prepared state (Figure 13a). The decrease of $R_{el}$ is explained by the grain growth and reduction of defects in the films during the heat treatment. It is concluded that all barrier layers were equally able to prevent the oxidation of the films in HV. After annealing, $R_{el}$ of the films with additional Al was not lower than that of the film without additional Al.

For the films on the CTGS substrate after annealing at 900 °C in HV, $R_{el}$ strongly depended on the applied barrier/cover layers (Figure 13b). As already observed in the cross-section images, only the two films with the combined cover/barrier layer were not oxidized. These two films reached the same low $R_{el}$ (17 μΩcm) as compared to the films on SiO${}_{2}$/Si. Both films with the SiO${}_{2}$ barrier behaved differently. The addition of Al layers led to a higher $R_{el}$ (33 μΩcm without, 50 μΩcm with Al layers). A stronger oxidation was also observed in the cross-section images. The highest $R_{el}$ was measured for the film with AlN barrier layers (70 μΩcm).

In the case of the LGS substrate after annealing at 900 °C in HV, for all films except those covered with AlN, which had a higher value of 61 μΩcm, a low resistivity comparable to the films on SiO${}_{2}$/Si was measured (18–21 μΩcm) (Figure 13c).

For all samples with additional Al layers, the annealing in air at 600 °C led to an increase of $R_{el}$. There were only slight differences between the different substrates. The highest value was reached for the (4)-AlN/SiO${}_{2}$/Al(7 nm) sample system (69–76 μΩcm), followed by (3)-AlN/SiO${}_{2}$/Al(10 nm) (56–63 μΩcm), (1)-AlN (52–56 μΩcm), and (2)-SiO${}_{2}$ (40–48 μΩcm). The lowest value was measured for the SiO${}_{2}$ covered film without additional Al (40–47 μΩcm). The lowest value for this sample can be explained by the formation of RuAl${}_{2}$ in the other samples at the interfaces between the Al and the RuAl, which is a semiconductor with a low electrical resistivity at RT [15].

Since after annealing at 800 °C in air, the films on LGS were mostly destroyed, the electrical resistivity after this heat treatment was only measured for the films on SiO${}_{2}$/Si and CTGS. Except for the (1)-AlN/Al sample system, all other films had a low electrical resistivity (19–24 μΩcm), which was only slightly higher than that measured for the HV annealed films. It was not possible to measure an electrical resistivity for the (1)-AlN/Al sample system on CTGS, and on SiO${}_{2}$, a higher value (36 μΩcm) than for the other samples was determined. For the (1)-AlN/Al sample, the cross-section images, AES, and XRD measurements indicated that the film had a thick Al${}_{2}$O${}_{3}$ layer on top and the lower region of the film consisted of Ru and RuAl grains, which were isolated by Al${}_{2}$O${}_{3}$ so that the films were not conductive despite the presence of metallic grains.

After annealing at 900 °C, all films were more or less destroyed, so that no electrical measurements were performed.

## 4. Conclusions

This paper reports on the further significant improvement of high-temperature stable RuAl thin films on the piezoelectric CTGS and LGS in comparison to reference thermally oxidized Si substrates. In former work for RuAl films on SiO${}_{2}$/Si, a high temperature stability up to 800 °C in air for 10 h was reached if a 20 nm thick SiO${}_{2}$ cover layer was applied. Now, the focus was on stabilizing the films against oxidation originating from the CTGS or LGS substrate.

In the case of the air annealed samples, the results showed that the films on CTGS were more stable than the films on LGS. On the other hand, in the case of annealing in HV, the films on LGS were less oxidized than the films on CTGS. In contrast to this, the results of our former work on RuAl thin films directly deposited on the substrates after annealing at 800 °C in HV showed a stronger oxidation for the films on LGS [10]. The opposite behavior observed in this work for the annealings at 900 °C in HV might originate from a different chemical reactivity between the substrates and the Al of the RuAl (set up in the former work) as compared to that between the substrate and the respective barrier material presented in this paper. Regarding the activation energy of the oxygen vacancy diffusion, it is known that in LGS, it is lower (0.8–1 eV) as compared to CTGS (1.3 eV), as, e.g., described by Zhang et al. [16]. However, a detailed explanation of the observed behavior cannot be given here, and further research by experts in the field of the high temperature stable crystals is needed.

A comparison of the cover layers revealed that a single AlN layer was least suited to protect RuAl thin films against oxidation. On SiO${}_{2}$/Si substrates, all barrier layers led to stable RuAl films in the case of HV annealing up to 900 °C, and except the film with AlN barrier layers, all films were stable during annealing in air up to 800 °C for at least 10 h. On CTGS, also except the film with AlN barrier layers, all other films were stable during annealing in air up to 800 °C. However, during annealing in HV, only the films with the combined barrier layers were not oxidized. In this case, the combination of both barrier layers was required to protect the RuAl film from oxygen, which diffused out of the substrate. The films on LGS were strongly oxidized already during annealing in air at 800 °C. During annealing in HV at 900 °C, all films except the AlN covered samples were stable.

In summary, we conclude that a combined barrier layer consisting of 20 nm AlN and 20 nm SiO${}_{2}$ is able to prevent the oxidation of the RuAl film on CTGS up to 800 °C in air and 900 °C in HV. On LGS, stable films are realized up to 600 °C in air and 900 °C in HV.

The addition of 10 nm Al layers on the top and bottom of the RuAl film results in an improved phase formation as was seen in the XRD measurements of the samples with the SiO${}_{2}$ cover with and without the addition of Al layers.

Comparing the two film architectures (10 nm Al)–(110 nm RuAl)–(10 nm Al) and (7 nm Al)–(55 nm RuAl)–(7 nm Al)–(55 nm RuAl)–(7 nm Al) revealed that after annealing in the latter films, the grains were larger and a texture with a tilt of the (100) axis of about 22° with respect to the film normal developed. In addition, it was found that at the interface to the substrate, a stronger chemical reaction with the substrate material took place for the (3)-AlN/SiO${}_{2}$/Al(10 nm) system, which was seen in the STEM images.

These results represent an important progress in the realization of a material system for high temperature SAW applications. The next step is the investigation of structured RuAl electrodes and the development of SAW devices based on the RuAl films with the combined barrier layer system on CTGS, which is the subject of our ongoing work.

## Abbreviations

The following abbreviations are used in this manuscript:

AES	Auger electron spectroscopy
as-dep.	as-deposited
EDX	energy dispersive X-ray spectroscopy
FIB	focused ion beam
HV	high vacuum
IDT	interdigital transducer
RT	room temperature
SAW	surface acoustic wave
SEM	scanning electron microscopy
TEM	transmission electron microscopy
STEM	scanning electron transmission microscopy
vdP	van der Pauw
XRD	X-ray diffraction
